# Benchmarking ensemble and deep learning models for drought-responsive gene prioritisation in rice using multi-omics data

**DOI:** 10.1371/journal.pone.0360052

**Published:** 2026-10-09

**Authors:** Kumanan N. Govaichelvan, Dharini Pathmanathan, Rabiatul Adawiah Zainal-Abidin, Arpah Abu

**Affiliations:** 1 Institute of Biological Sciences, Faculty of Science, Universiti Malaya, Kuala Lumpur, Wilayah Persekutuan Kuala Lumpur, Malaysia; 2 Institute of Mathematical Sciences, Faculty of Science, Universiti Malaya, Kuala Lumpur, Wilayah Persekutuan Kuala Lumpur, Malaysia; 3 Biotechnology and Nanotechnology Research Centre, Malaysian Agricultural Research and Development Institute (MARDI), Serdang, Selangor, Malaysia; 4 Centre of Research in Systems Biology, Structural Bioinformatics and Human Digital Imaging, Universiti Malaya, Kuala Lumpur, Malaysia; Texas A&M University, UNITED STATES OF AMERICA

## Abstract

Identifying drought-responsive genes is critical for climate-resilient crop breeding, yet computational gene–trait association often relies on single-omics or generalized genomic datasets. Optimal algorithm selection for high-dimensional, small multi-omics datasets also remains a persistent challenge. We present a machine learning framework that extends the QTG-Finder feature set by integrating transcriptomic and proteomic features to classify drought-responsive genes in rice. Using MBKBase annotations, we evaluated the ability of these models to prioritise 336 drought-responsive genes among 2,416 abiotic stress-associated genes, of which the remaining 2,080 constituted an operational background set. We tested four algorithms: Random Forest, XGBoost, one-dimensional convolutional neural network, and multilayer perceptron, while addressing severe class imbalance and assessing model stability. Tree-based ensemble methods achieved higher performance than the deep learning models in this dataset, which showed early overfitting. The fine-tuned Random Forest achieved a recall of 0.67 and an AUC-ROC of 0.87 under nested cross-validation, conditional on a feature matrix assembled before gene-level data splitting. Across repeated evaluations of a small internal holdout of 10 genes, mean accuracy and recall were 0.90 and 0.63; this set is too small to support a generalizability claim and is reported only as a consistency check. The newly added transcriptomic and proteomic features improved recall from 0.47 to 0.67 compared with genomics alone. Feature importance and ablation analyses indicated that transcriptomics-derived features, particularly measures of expression distribution, magnitude, and direction, contributed most to predictive performance. Functional enrichment analysis supported the biological relevance of the prioritised genes. These results demonstrate the value of multi-omics integration for gene prioritisation in small tabular datasets while highlighting current limitations of deep learning in this setting.

## Introduction

Rice (*Oryza sativa*) is a staple crop in many regions of the world, accounting for 50% of the energy supply for the global population [1]. Therefore, ensuring a stable rice supply is critical for maintaining the food security and economic stability in many countries [2]. However, drought stress, affecting more than one-third of the cultivated area across the world, poses a significant threats to rice production [3].

Drought stress in plants is characterized by decreased water content, reduced leaf water potential, decreased turgor pressure, diminished stomatal activity, and decreased cell expansion and proliferation. Notably, specific genotypes within the rice species that can withstand sub-optimal growth conditions are identified as ‘stress-tolerant’. Hence, ‘drought tolerance’ is defined as the plant’s ability to maintain survival and productivity under low tissue water content. Plants have evolved to withstand external stress factors to sustain their metabolic homeostasis through various mechanisms, including alterations of cellular organelles, metabolic pathways, phytohormonal composition, physiological and morphological processes [4].

Consequently, the response to drought stress is determined by genetic complexity and environmental factors, making it a quantitative trait. Its genetic complexity arises from the involvement of multiple quantitative trait loci (QTL) and their non-additive interactions [5]. Sequence variation within genes located in QTL regions can lead to phenotypic diversity. Moreover, understanding gene-trait relationships helps scientists to interpret evolutionary forces and molecular mechanisms that contribute to the genetic architectures of adaptive traits [6]. Two widely used approaches for QTL identification are QTL linkage mapping and genome-wide association study (GWAS), each with its own strengths and limitations [7]. However, identifying causal genes remains challenging as many QTLs often encompass numerous candidate genes. While conventional fine mapping is a reliable strategy, it is also expensive, time-consuming, and labour-intensive [5]. Meanwhile, drought response in rice involves complex physiological adaptations regulated by multiple interconnected genetic pathways, creating a computational bottleneck for identifying drought-responsive genes.

To address these limitations, computational approaches offer scalable alternatives for candidate gene prioritisation. In recent years, machine learning (ML) has emerged as a promising tool for solving complex biological problems. Leveraging data generated from high-throughput experiments, studies have shown that ML models have the ability to prioritise candidate genes associated with specific traits [8–10]. QTG-Finder [5], a recent example, was developed to predict rice QTLs that are highly associated with abiotic and biotic stress, using genomics features derived from paralog copy number, gene ontology (GO), and the number of SNPs. Then, GRAiN [11] was developed to predict rice transcription factors associated with drought-response, by constructing network features from transcriptomics data only. Aforementioned computational tools such as QTG-Finder and GRAiN have made progress in gene prioritisation, they primarily rely on limited feature types, predominantly genomic variants or co-expression networks. Numerous ML-based gene prioritisation methods have been proposed; however, their performance depends strongly on feature composition and data characteristics [12], particularly in curated multi-omics datasets with relatively small sample sizes [13].

Subsequently, integration of data from multiple omics was observed to reduce false positives [14]. Likewise, the performance of gene prediction improved [15] with the QTG-Finder dataset, upon the addition of 12 new features from protein–protein interaction (PPI), gene structure, and expression variation. These studies highlight the need for the exploration and evaluation of new features for the integration of multiple omics gene-traits analysis. Likewise, this study intends to extend the framework by introducing drought-specific features for drought-responsive gene classification.

Despite ML enabling an integrative approach, developing an ML model requires comprehensive evaluation of the selected algorithm, processed features, and evaluation techniques [16–18]. A study involving six species and 18 traits found no single algorithm performed optimally across all species, highlighting the importance of matching algorithmic and genetic architectures [19]. Hence, selecting a suitable algorithm is critical for predictive model development, as species and trait-specific approaches offer useful insights.

Ensemble and deep learning approaches have both shown strong performance in classification tasks, but their relative suitability depends on data size and structure, with tree-based ensembles often performing better in small, high-dimensional datasets typical of curated multi-omics studies [20,21]. The construction of EL algorithms involves the aggregation of multiple algorithms to enhance predictive accuracy [22]. In contrast, DL algorithm offers automatic feature extraction through the construction of networks and edges; though often, it is computationally intensive [18]. However, their comparative effectiveness for classifying drought-responsive genes using integrated multi-omics features remains limited.

This study presents a multi-omics classification framework to prioritise drought-responsive genes in rice using 44 integrated features derived from genomics, transcriptomics, and proteomics data. We benchmark Random Forest, XGBoost, multilayer perceptron, and one-dimensional convolutional neural network models using repeated nested cross-validation and stability analysis. Feature importance and ablation studies were performed to quantify the contribution of different omics layers to predictive performance, and functional enrichment analysis was used to assess the biological relevance of the prioritised genes. The classification performance was then tested with manually created holdout set of genes, as this is still an exploratory phase, and obtaining similar set of data is currently unfeasible.

## Methods

Fig 1 shows the workflow used to develop a classification model for predicting genes associated with drought response. The process includes dataset preparation, model development and model evaluation, each of which is explained in the following subsections.

**Fig 1:**
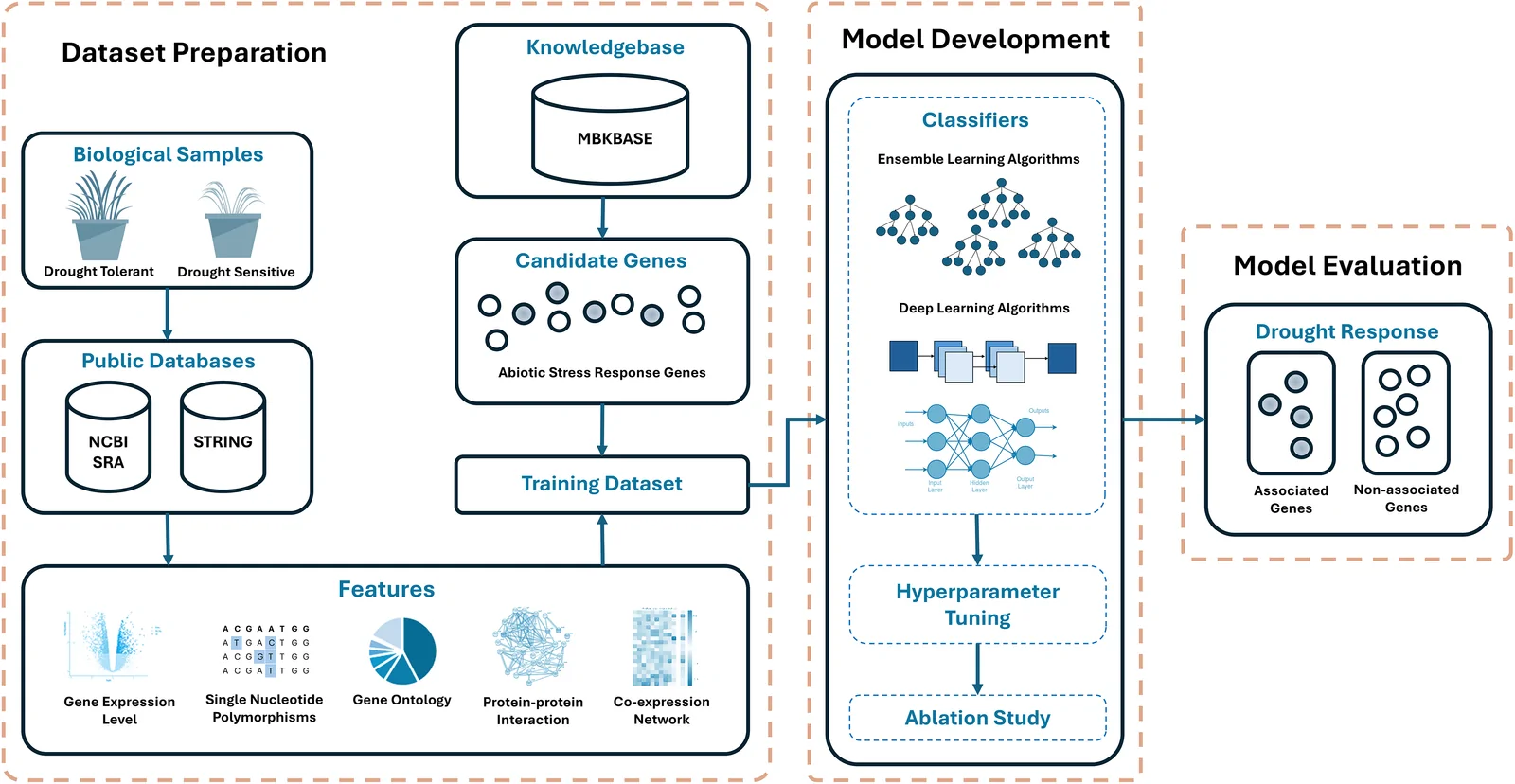
Workflow of classification model development.

### Dataset preparation

MBKBase (https://mbkbase.org/rice/knownGene) [23] was used as the reference database to annotate stress response genes, including those specifically associated with drought response. Out of 2416 identified abiotic stress response genes, 336 were labelled as drought-responsive in MBKBase. The remaining 2080 genes were treated as an operational background set. Gene labels indicating ‘associated’ or ‘non-associated’ with drought were obtained from the KnownGene module.The KnownGene module in MBKBASE provides the most recent functional annotations of experimentally validated alleles compiled from sources such as funRiceGenes, GenBank, Oryzabase and Q-Taro. However, these gene labels may change as new high-throughput studies become available. Drought-responsive genes represent a subset of abiotic stress response genes [24]. Hence, this study narrows the scope of ML on prioritising genes specifically associated with drought response from the broader abiotic stress context.

Subsequently, to implement the ML model, a total of 44 features were used. 28 from genomics, 14 from transcriptomics and two from proteomics (S1 Table). The methods used to generate these features are explained below.

### Genomic features

Genomic features were obtained from publicly available repositories and publications. The QTG-Finder [5] repository provided 27 features used to rank genes associated with abiotic and biotic stress response. This dataset incorporates seven single nucleotide polymorphism features retrieved from Rice-SNP-Seek [25], 17 functional annotation features based on Gene Ontology, one co-function network descriptor from RiceNet [26] and two evolutionary features from [27]. Additionally, as the 28th feature, genes were labelled as expression quantitative trait locus (eQTL) based on [28] due to their role in response to abiotic stress factors.

### Proteomics features

Protein-protein interactions (PPI) reflect gene interactions, based on the involvement of the encoded proteins in a specific biochemical pathway. A higher score correlates with stronger interactions [15]. PPI data for Oryza sativa was obtained from STRING-DB (version 12.0). Two features were extracted from the database, namely *PPI_Counts_400* and *PPI_Counts_700*, which represent the number of interactions for each gene, where the score is above 400 and 700 respectively.

### Transcriptomics features

Transcriptomic features were derived from RNA-seq data. The RNA-seq data deposited in the Sequence Read Archive (SRA NCBI) were manually searched to categorize drought-related experiments performed on *Oryza sativa*. Sequence accessions were obtained from these BioProjects. PRJNA248474, PRJNA420056, PRJNA608550, PRJNA608552, PRJNA667047. In total, the selected studies included 42 drought-sensitive samples and 48 drought-tolerant samples. Studies were selected based on the following criteria.

- availability of a corresponding peer-reviewed publication;
- a minimum of two biological replicates per condition.
- inclusion of both drought-tolerant and drought-sensitive rice varieties.
- complete and well-annotated metadata in the public repository.
- generation of paired-end sequencing data.

A standardized pipeline was established to convert the raw sequencing data into gene counts. The raw data were downloaded as FASTQ files using *SRAToolkit* (V2.8.2). *FastQC* [29] was used to perform quality checks. Adapters and low-quality bases were trimmed using *Trimmomatic* (V0.36) [30]. High quality reads were retained and aligned to the reference genome sequence (IRGSP-1.0) using *HISAT2* [31]. *HISAT2* resulted in BAM files which were then sorted and quantified using *featurecounts* [32]. Each experiment was processed separately and subsequently combined for feature generation.

To eliminate batch effects arising from experiments conducted in different laboratories, *ComBat-seq* [33] from the *SVA* package [34] in R was employed. This method applies a negative binomial regression model that preserves the integer nature of RNA-seq count data, ensuring compatibility with standard differential expression tools that require integer input [35]. *ComBat-seq* was selected for its demonstrated robustness in correcting unwanted variation during integrative RNA-seq analyses, as supported by previous studies [36,37]. Principal component analysis (PCA) was performed to examine whether the samples showed apparent group separation in the reduced-dimensional space (S1 Fig).

Then, the gene counts from tolerant and sensitive varieties were grouped and processed separately with the *DESeq2* [38] package in R to generate features based on differential gene expression. Four features were extracted using *DESeq2*. basemean, *log2-fold-change*, significant upregulation, and significant downregulation, for both tolerant and sensitive varieties, respectively. Significance thresholds were established to determine upregulated and downregulated genes. genes were considered significantly upregulated if the *log2-fold-change* exceeded 1.5 with an *adjusted p-value* below 0.05, and significantly downregulated if the *log2-fold-change* was below −1.5 with an *adjusted p-value* below 0.05. A total of eight differential expression (DE) features were generated.

Weighted Gene Co-expression Network Analysis (WGCNA) is a systems biology method that facilitates the dissection of the underlying biological mechanisms for a co-expressed gene set [39]. WGCNA shows correlated genes among modules associated with a specific trait or observation, which may share important biological regulatory roles [40]. From WGCNA, three features were derived, each individually processed for both tolerant and sensitive varieties, resulting in a total of six co-expression (CE) features.

It should be noted that all differential expression and co-expression feature extraction steps were performed globally across the entire processed RNA-seq dataset prior to dataset splitting and cross-validation fold generation. These transcriptomic features were constructed without using the gene-level MBKBASE drought-association labels. Nevertheless, because feature construction preceded gene-level splitting, genes subsequently held out contributed to the estimation of parts of the fixed feature representation.

In total, 17 new features were added to the QTG-Finder dataset, to repurpose it for the classification of genes associated with drought response. The newly added features are listed and described in Table 1.

**Table 1:** List of newly generated features.

No.	Features	Descriptions
1.	*Basemean_Tol*	Average of the normalized count values, divided by size factors, taken from drought tolerant samples.
2.	*Log2FC_DESeq2_Tol*	Magnitude of change in gene expression level in tolerant samples.
3.	*Up_Tol*	Upregulated genes in tolerant samples.
4.	*Dn_Tol*	Downregulated genes in tolerant samples.
5.	*Basemean_Sen*	Average of the normalized count values, divided by size factors, taken from drought sensitive samples.
6.	*Log2FC_DESeq2_Sen*	Magnitude of change in gene expression level in sensitive samples.
7.	*Up_Sen*	Upregulated genes in sensitive samples.
8.	*Dn_Sen*	Downregulated genes in sensitive samples.
9.	*Mod_No_Tol*	WGCNA module numbers generated using tolerant samples.
10.	*Mod_High_Corr_Tol*	Highly correlated modules with drought response in tolerant samples.
11.	*Hub_Genes_Tol*	Genes from tolerant samples belonging to module correlation >0.5, and achieves GS > 0.3 and MM > 0.7
12.	*Mod_No_Sen*	WGCNA module numbers generated using sensitive samples.
13.	*Mod_High_Corr_Sen*	Highly correlated modules with drought response in sensitive samples.
14.	*Hub_Genes_Sen*	Genes from sensitive samples belonging to module correlation >0.5, and achieves GS > 0.4 and MM > 0.8
15.	*PPI_counts_400*	Number of genes with PPI scores above 400, reflecting moderate connection.
16.	*PPI_counts_700*	Number of genes with PPI scores above 700, reflecting strong connection
17.	*eQTL*	Genes belonging to expression quantitative trait loci group.

### Data balancing

Upon generating the dataset with all features, the proportion of ‘associated’ and ‘non-associated’ genes was evaluated. The ‘non-associated’ genes were excluded based on *DES*eq2 *adjusted p-values* obtained from drought tolerant and susceptible rice varieties. Lower *adjusted p-values* reflect greater significance in gene dysregulation following drought treatment [41]. Filtering genes based on their level of differential expression is a recommended strategy for optimizing data mining [8]. As a result, the ‘non-associated’ records in the datasets were excluded based on the increasingly stringent *adjusted p-values* of 1 × 10 **^−^**^1^, 1 × 10 **^−^** ^2^, 1 × 10 **^−^** ^3^, 1 × 10 **^−^** ^4^ and 1 × 10 **^−^** ^5^, at 10-fold decrements. Table 2 shows the different proportion of associated and non-associated genes after the removal of ‘non-associated’ genes.

**Table 2:** Description of Dataset Dimensions.

Dataset	*Adjusted p-values*	Total Genes	Non-associated %	Associated %
Dataset 1 (DS1)	–	2416	86.1	13.9
Dataset 2 (DS2)	1 × 10 **^−^** ¹	1609	79.9	20.1
Dataset 3 (DS3)	1 × 10 **^−^** ²	1231	72.7	27.3
Dataset 4 (DS4)	1 × 10 **^−^** ³	1019	67	33.0
Dataset 5 (DS5)	1 × 10 **^−^** ⁴	877	61.7	38.3
Dataset 6 (DS6)	1 × 10 **^−^** ^5^	786	57.3	42.7

The baseline dataset, referred to as Dataset 1 (*S1 Data*), contained 86.1% ‘non-associated’ (N.As.) and 13.9% ‘associated’ (As.) genes, including all abiotic stress-responsive genes from MBKBASE. Severe class imbalance necessitated the derivation of five additional datasets with reduced ‘non-associated’ genes, guided by *adjusted p-values* from *DES*eq2. Only Dataset 6 achieved near balance, as shown in Table 2, while the Datasets 2–5 still showed imbalance. As an internal consistency check, a small holdout set was created by randomly isolating five ‘associated’ and five ‘non-associated’ genes. Since these genes were drawn from the same pool and share the globally constructed transcriptomic feature representation, this set is not an independent validation cohort, and its size (n = 10) precludes inference about generalizability to unseen genes.

### Model development

The study employed four supervised ML algorithms for classification, two EL algorithms. Random Forest (RF) and XGBoost (XGB), and two DL algorithms. 1-Dimensional Convolutional Neural Network (1D-CNN) and Multilayer Perceptron (MLP).

The EL algorithms were implemented using the *scikit-learn* library, while DL models were developed using the *TensorFlow* and *Keras* libraries. Initial architecture and hyperparameters were selected based on the approach described by [12], with systematic optimization conducted on Dataset 6 before application to multiple datasets. Various configurations of dense, convolutional, and max-pooling layers, as well as regularization parameters and dropout layers, were evaluated to determine optimal performance.

The selected 1D-CNN architecture consisted of a convolutional layer with 64 filters and a kernel size of 3, using ReLU activation and accepting input with the shape of training features. ReLU activation was chosen due to its ability to introduce non-linearity into the model while avoiding the vanishing gradient problem, thus improving training efficiency and performance [42]. This was followed by a max-pooling layer with a pool size of 2 to reduce dimensionality, and a dropout layer (rate = 0.2) to prevent overfitting. The features were then flattened and passed through a dense layer with 64 neurons and ReLU activation, followed by another dropout layer (rate = 0.2). The final dense layer, the output layer, used Sigmoid activation, as it is deemed suitable for binary classification [43].

For the MLP model, a sequential architecture was implemented with three hidden layers. The first layer contained 64 neurons with ReLU activation and L2 regularization, followed by a dropout layer (rate = 0.2). The second hidden layer expanded to 128 neurons with ReLU activation and L2 regularization, also followed by a dropout layer (rate = 0.2). The third hidden layer was reduced back to 64 neurons with ReLU activation and L2 regularization. Finally, the output layer consisted of a single neuron with sigmoid activation for binary classification. Both DL models were compiled with the Adam optimizer, binary cross-entropy loss function, and accuracy as the training metric for optimization. Models were trained for 10 epochs with a batch size of 32 for performance evaluation. To examine learning behaviour and convergence, separate diagnostic runs with extended training up to 50 epochs were conducted to generate training and validation loss curves.

In contrast, hyperparameters for EL algorithms were fine-tuned after applying them onto the Dataset 6 to ensure the suitability of the algorithms first. A paired t-test was performed to check if there is a significant improvement due to hyperparameter tuning.

Additionally, simpler baseline models were also tested, which includes Logistic Regression, Support Vector Machines, K-Nearest Neighbour and Naïve Bayes. However, results of baseline models were omitted due to poor performance.

### Model evaluation

The models were evaluated using a nested cross-validation (NCV) framework, with performance assessed through metrics including accuracy, precision, recall, F1 score, and Area Under Curve – Receiver Operating Curve (AUC-ROC). NCV, with an inner loop for model selection and an outer loop for evaluation, reduces overfitting risks and provides more reliable performance estimates [44]. The inner loop was set to three-folds, whereas the outer loop was set to five-folds. Accuracy reflects the percentage of correctly classified data, while precision measures the percentage of correctly classified positive instances relative to all instances classified as positive. Recall denotes the ratio of positively classified instances to all actual positive instances, and the F1 score provides a balanced assessment of precision and recall. AUC-ROC evaluates the model’s ability to distinguish between classes, with values closer to 1 indicating better performance. Disparity between precision and recall indicates bias in performance due to class imbalance, which lowers F1 score [45].

The NCV was employed to limit leakage during model evaluation, but the scheme operates on a feature matrix assembled before gene-level splitting. The reported metrics are therefore estimates of classifier performance conditional on this fixed feature representation, rather than estimates of the end-to-end pipeline comprising feature construction and classification. All performance values reported in this study should be interpreted in that restricted sense.

To ensure reliability, the models underwent 20 repetitions of training, with adjustments to the *random.seed* number each time. This iterative approach mitigates the impact of randomness inherent in ML algorithms on model performance [46]. The performance measures reported is the average of 20 repetitions and along with its standard deviation. Standard deviation quantifies the average deviation of performance measures from their mean, offering insight into the spread around the mean value.

Performance stability was further assessed using three evaluation methods namely nested cross-validation method (NCV), the holdout method and K-fold cross-validation. A stable model is expected to produce results with minimal discrepancy across these different evaluation methods [47,48].

A sensitivity analysis was performed using 10 alternative datasets, each assembled by randomly sampling “non-associated genes” from the remaining non-associated pool, while completely excluding the original Dataset 6 negatives. Each dataset maintained the class proportions of Dataset 6.

### Feature importance analysis

Upon identifying suitable algorithms, feature importance scores were obtained using wrapper methods. These scores were averaged across 20 repetitions with different random seeds to ensure result stability. Feature importance was assessed both before and after hyperparameter tuning to evaluate the impact of optimization on feature selection.

Following the identification of important features, an ablation study was conducted to systematically evaluate the contribution of different omics components to model performance. In this study, omics components (DE, CE, PPI, and genomic features) were iteratively removed from the feature set, and models were retrained and evaluated using the same performance metrics. This approach allowed for the assessment of each omics component’s relative contribution to the model’s predictive power, providing insights into which data types were most informative for identifying drought-responsive genes.

### Functional enrichment analysis

Based on the suitable hyperparameters for the best performing classification algorithm, the genes are ranked using classifier generated probabilistic scoring. ShinyGO (http://bioinformatics.sdstate.edu/go/) [49] was used for the Gene Ontology (GO) terms to retrieve biological process (BP) annotations of the top 5% ranked genes. A statistical threshold (FDR ≤ 0.05) was applied to identify functional annotations significantly enriched by the queried genes.

## Results

### Multi-omics Dataset

Dataset preparation is a crucial step in developing ML models to address complex questions. Features should be representative to enable supervised ML algorithms to distinguish between labels [16]. A total of 90 RNA-seq samples (1.4 TB) were processed to generate gene counts data, and 44 features were derived from genomics, transcriptomics, and proteomics data (Table 1).

### Model performance

Four models were used to classify drought-associated genes based on 44 multi-omics features. The best performance was achieved with Dataset 6 (Fig 2) (S2 Table), shows the importance of using balanced datasets. In contrast, Datasets 1–5 showed high precision but low recall due to class imbalance, which led to the misclassification of minority class instances [50]. A threshold value of 0.60 for performance measures is indicated by the red line in the following figures.

**Fig 2:**
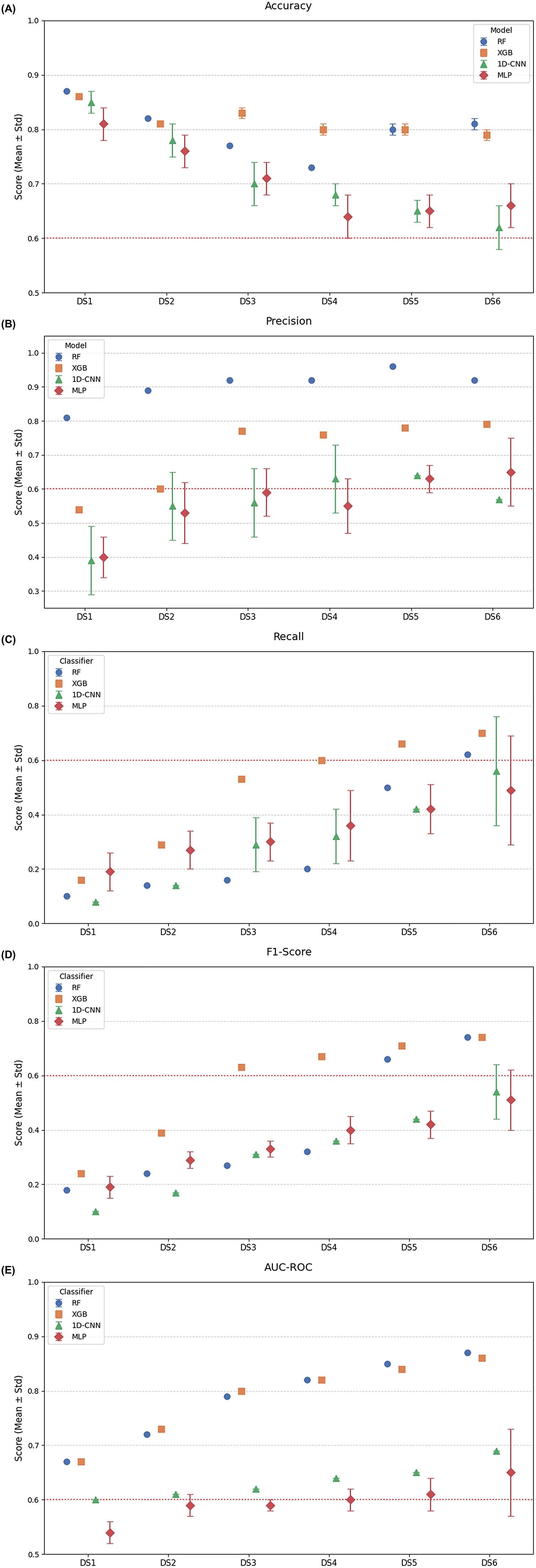
Performance measures of machine learning algorithms on six different datasets (DS1-DS6) using nested cross-validation. (A) Accuracy. (B) Precision. (C) Recall. (D) F1-Score. (E) AUC-ROC.

RF and XGB consistently performed well, achieving scores above 0.60 across all performance measures, indicating their suitability for gene classification. Tree-based ensemble learning (EL) methods effectively handled dataset complexity, as previously reported [11,19]. Based on NCV, RF and XGB demonstrated robust classification performance on Dataset 6, with recall values of 0.62 and 0.70, respectively, and both achieving AUC-ROC above 0.86 (S2 Table). The superior recall of XGB suggests greater sensitivity in identifying ‘associated’ genes, likely due to its built-in regularization, which prevents overfitting by controlling tree complexity and reducing variance, as observed in previous studies [51]. RF, on the other hand, leverages the aggregation of multiple decision trees, making it inherently resistant to overfitting by averaging predictions and reducing bias [52]. The consistent performance of these ensemble models highlights their ability to handle complex, multi-omics datasets, unlike CNN and MLP, which struggled with feature extraction and learning stability. These findings suggest that EL methods are better suited for datasets of this size and complexity, as they effectively mitigate the risk of overfitting, which is often exacerbated in smaller datasets [13].

Conversely, the 1D-CNN and MLP underperformed, with recall values falling below 0.60, indicating a high rate of false negatives, despite the use of a balanced dataset. Recall is particularly important as it measures correctly classified ‘associated’ genes [53]. Limited dataset size likely constrained deep learning models’ generalizability, as reported in previous studies [51,54]. Training-validation loss plots over 50 epochs revealed early overfitting in 1D-CNN (Fig 3A), while MLP exhibited underfitting by slow learning progress (Fig 3B). The 1D-CNN showed early overfitting based on the training–validation loss curves exhibiting difficulty in extracting relevant features from the data [55]. In contrast, the MLP model showed limited performance improvement across epochs, indicating underfitting under the evaluated configuration [56]. These observations further reinforce the suitability of tree-based models for handling complex, multi-omics datasets.

**Fig 3:**
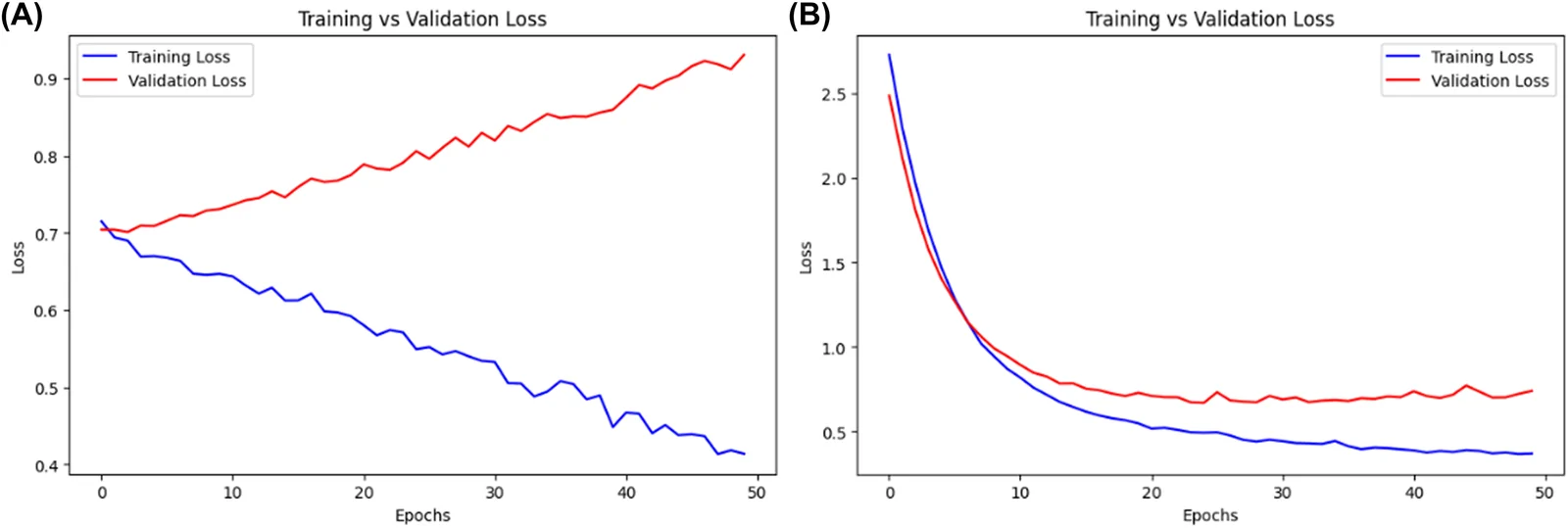
Training and Validation Loss Plot of (A) 1-Dimensional Convolutional Neural Network and (B) Multi-layer Perceptron. The 1D-CNN showed early overfitting, while MLP showed underfitting across 50 epochs.

Stability analysis using three evaluation approaches (Table 3) showed that the tree-based models, RF and XGB, exhibited minimal performance fluctuations, while the DL models showed notable instability. A similar observation was reported [48], where tree-based model demonstrated consistent performance across multiple evaluation approaches, particularly with small dataset. Meanwhile, the F1-score of 1D-CNN varied significantly across approaches. 0.54 ± 0.11 (NCV), 0.74 ± 0.01 (Holdout), and 0.57 ± 0.10 (CV). Despite hyperparameter tuning, the DL models continued to underperform, underscoring the higher within-dataset performance and stability of RF and XGB. XGB’s built-in regularization helped mitigate overfitting, while RF’s ensemble architecture made it inherently more resilient to noise and variance [54]. The findings suggest that EL methods are better suited for this dataset, offering higher discriminative performance and stability under the evaluated fixed-feature setting.

**Table 3:** Performance stability of ML algorithms tested with three different evaluation approaches.

Algorithm	Performance Measures	Nested cross-validation	80.20 Split	K-fold Cross-validation (K = 5)
		Mean ± SD	Mean ± SD	Mean ± SD
**RF**	Accuracy	0.81 ± 0.01	0.82 ± 0.00	0.82 ± 0.03
	Precision	0.92 ± 0.01	0.91 ± 0.01	0.91 ± 0.06
	Recall	0.62 ± 0.02	0.63 ± 0.01	0.64 ± 0.07
	F1-Score	0.74 ± 0.01	0.74 ± 0.01	0.75 ± 0.05
	AUC- ROC	0.87 ± 0.00	0.86 ± 0.00	0.87 ± 0.02
**XGB**	Accuracy	0.79 ± 0.01	0.77 ± 0.00	0.79 ± 0.02
	Precision	0.79 ± 0.01	0.75 ± 0.00	0.80 ± 0.06
	Recall	0.70 ± 0.01	0.69 ± 0.00	0.71 ± 0.02
	F1-Score	0.74 ± 0.01	0.72 ± 0.00	0.75 ± 0.03
	AUC- ROC	0.86 ± 0.01	0.85 ± 0.00	0.85 ± 0.02
**1D-CNN**	Accuracy	0.62 ± 0.04	0.81 ± 0.01	0.67 ± 0.03
	Precision	0.57 ± 0.06	0.92 ± 0.02	0.64 ± 0.08
	Recall	0.56 ± 0.20	0.62 ± 0.02	0.53 ± 0.28
	F1-Score	0.54 ± 0.11	0.74 ± 0.01	0.57 ± 0.10
	AUC- ROC	0.69 ± 0.02	0.79 ± 0.01	0.70 ± 0.06
**MLP**	Accuracy	0.64 ± 0.01	0.63 ± 0.10	0.62 ± 0.04
	Precision	0.60 ± 0.01	0.62 ± 0.04	0.59 ± 0.12
	Recall	0.50 ± 0.10	0.57 ± 0.09	0.44 ± 0.22
	F1-Score	0.52 ± 0.05	0.52 ± 0.04	0.46 ± 0.13
	AUC- ROC	0.64 ± 0.02	0.66 ± 0.04	0.61 ± 0.06

Note. RF = Random Forest, XGB = XGBoost, 1D-CNN = 1-Dimensional Convolutional Neural Network, MLP = Multilayer Perceptron

### Hyperparameter tuning

Despite *GridSearchCV* is commonly used for hyperparameter optimization within the NCV framework, it was not adopted in this study. This is because *GridSearchCV* selects hyperparameters based on performance tied to a specific *random.seed* which can bias the results [48]. As a result, a manual grid search was performed within the two-tiered nested approach, with tested parameters as listed in Table 4. The optimal parameters were identified by averaging performance measures over 20 iterations. The combination that yielded the highest mean values in accuracy, F1-score, and AUC-ROC is presented in Table 4.

**Table 4:** Hyperparameters used for tuning the XGBoost (XGB) and Random Forest (RF) models.

Algorithm	Hyperparameters	Default Parameters	Best Parameters	Tested Range
XGB	n_estimators	100	100	100, 300, 500
	max_depth	3	9	3, 6, 9
	learning_rate	0.3	0.05	0.05, 0.1, 0.3, 0.5
	subsample	1	0.7	0.5, 0.7, 1.0
	reg_lambda	1	5	0.5, 1, 5
RF	n_estimators	100	300	100, 300, 500
	max_depth	3	20	3, 5, 10, 20
	min_samples_split	2	10	2, 5, 10
	min_samples_leaf	1	1	2, 1
	max_features	sqrt	sqrt	sqrt

To validate the effectiveness of hyperparameter tuning, a *t-test* was conducted to compare the performance of fine-tuned models against their default parameter settings. The results indicated that fine-tuned XGB and RF models achieved significantly higher overall performance. Although accuracy, F1-score, and AUC-ROC improved for XGB, recall slightly decreased from 0.70 to 0.67. In contrast, the fine-tuned RF model outperformed XGB, with a notable increase in recall (Table 5).

**Table 5:** The Performance of Ensemble Algorithms with Default and Fine-tuned Parameters are Compared with T-Test.

Algorithm	Performance Measures	Hyperparameters		
		Default	Fine-tuned	
		Mean ± SD	Mean ± SD	Direction
**XGB**	Accuracy	0.79 ± 0.01	0.81 ± 0.01*	⇧
	Precision	0.79 ± 0.01	0.86 ± 0.01*	⇧
	Recall	0.70 ± 0.01	0.67 ± 0.01*	⇩
	F1 Score	0.74 ± 0.01	0.75 ± 0.01*	⇧
	AUC-ROC	0.86 ± 0.01	0.86 ± 0.01*	⇧
**RF**	Accuracy	0.81 ± 0.01	0.82 ± 0.01*	⇧
	Precision	0.92 ± 0.01	0.89 ± 0.01*	⇩
	Recall	0.62 ± 0.02	0.67 ± 0.01*	⇧
	F1 Score	0.74 ± 0.01	0.76 ± 0.01*	⇧
	AUC-ROC	0.87 ± 0.00	0.87 ± 0.00*	⇧

Note. Values with an asterisk (*) show a significant difference with paired t-test at p-value < 0.05. Refer to S3 Table for the exact numerical values of t-test calculations.

The reduction in XGB’s recall may be attributed to the lower learning rate and subsample ratio, combined with an increased *max_depth*, which can elevate the risk of overfitting [57]. However, this effect was counterbalanced by a higher value for the lambda regularization parameter, making the model more conservative [58]. In RF, although increasing *max_depth* can also lead to overfitting [59], the addition of more trees and a higher minimum sample split threshold improved generalization [60]. The *max_features* parameter was set to ‘sqrt’, allowing the algorithm to automatically determine the optimal number of features at each split [61]. Further analysis of feature contributions is recommended to better understand the impact.

Across repeated evaluations of the small internal holdout, mean accuracy, recall and AUC-ROC were 0.90, 0.63 and 1.00, respectively. Given the holdout size of only five associated and five operational-background genes, these estimates are highly uncertain. They are reported only as an internal consistency check and neither establish generalizability nor distinguish sample-size effects from overfitting. Furthermore, the resampled datasets used for sensitivity analysis achieved accuracy, precision, recall and AUC-ROC of 0.89, 0.96, 0.76 and 0.93 respectively. These results indicate that the observed classifier performance was not unique to the particular negative sample used in Dataset 6. This analysis varies only the composition of the negative class; it does not quantify possible information sharing arising from global feature construction.

### Feature importance analysis

Feature importance was assessed for both RF and XGB before and after hyperparameter tuning (S4 Table). In RF, the most influential features primarily included the distribution, magnitude, and direction of gene expression derived from RNA-seq data in tolerant and sensitive samples, along with *PPI_Counts_400* and *network_weight* features. Following fine-tuning, *PPI_Counts_700* replaced *paralog_copy_number* in the importance ranking, although the latter remained within the top 15 features.

For XGB, the features *is_inorganic_nutrients_metabolism* and *hub_genes_dr_Sen* were consistently ranked among the top 10, along with other features also prioritised by RF. However, several features including *is_redox_metabolism*, *is_receptor*, *is_start_lost*, *is_stop_lost*, and *is_splice_site_SNP*, were completely disregarded by XGB, receiving zero importance scores across all 20 iterations. After hyperparameter tuning, an additional five features. *mod_high_corr_drought_Tol*, *hub_genes_dr_Tol*, *is_nucleotides_metabolism*, *is_fatty_acids_lipids_metabolism*, and *is_cofactors_metabolism*, were also excluded from the model. These results suggest that the fine-tuned RF model is less prone to noise and can make accurate predictions without entirely discarding any input features.

### Ablation study

The ablation analysis for RF (Table 6) revealed that removing any feature group resulted in a decline in model performance, with the most significant drop observed when all features except genomics were excluded. The complete model comprising DE, CE, PPI, and genomics achieved the highest accuracy (0.83) and AUC-ROC (0.87), highlighting the importance of multi-omics integration. Removing CE or PPI alone had minimal impact, however, excluding DE led to a more noticeable decline in performance. The removal of both DE and CE further reduced recall compared to excluding DE alone. Notably, the lowest recall (0.23) was observed when only CE feature was used, suggesting its limited standalone predictive power. While CE may not be effective on its own for gene classification, it contributes meaningfully in combination with other features. These results suggest that DE contribute significantly to model robustness. The findings are consistent with the feature importance analysis, indicating that DE-derived features were highly informative for model predictions.

**Table 6:** Ablation Study on Random Forest.

Features				Accuracy	Precision	Recall	F1-Score	AUC-ROC
Transcriptomics		PPI	Genomics					
**DE**	**CE**							
✔	✔	✔	✔	0.83 ± 0.02	0.88 ± 0.03	0.67 ± 0.06	0.76 ± 0.04	0.87 ± 0.03
–	✔	✔	✔	0.71 ± 0.03	0.70 ± 0.07	**0.55 ± 0.07**	0.61 ± 0.06	**0.76 ± 0.03**
✔	–	✔	✔	0.82 ± 0.02	0.87 ± 0.04	0.67 ± 0.05	0.76 ± 0.04	0.87 ± 0.03
–	–	✔	✔	0.67 ± 0.04	0.65 ± 0.09	**0.45 ± 0.08**	0.52 ± 0.07	**0.71 ± 0.05**
✔	✔	–	✔	0.82 ± 0.02	0.88 ± 0.04	0.67 ± 0.06	0.76 ± 0.04	0.86 ± 0.03
–	–	–	✔	0.66 ± 0.04	0.63 ± 0.08	**0.47 ± 0.06**	0.54 ± 0.06	**0.71 ± 0.04**
✔	✔	✔	–	0.81 ± 0.02	0.84 ± 0.04	0.67 ± 0.05	0.75 ± 0.04	0.85 ± 0.03
✔	✔	–	–	0.81 ± 0.02	0.84 ± 0.04	0.67 ± 0.05	0.74 ± 0.04	0.84 ± 0.03
✔	–	–	–	0.81 ± 0.02	0.84 ± 0.05	0.66 ± 0.05	0.74 ± 0.04	0.84 ± 0.03
–	✔	–	–	0.65 ± 0.04	0.76 ± 0.11	**0.23 ± 0.05**	0.35 ± 0.07	**0.64 ± 0.04**

Note. DE = Differential Expression, CE = Co-Expression, PPI = Protein-protein Interaction

### Functional enrichment analysis

As shown in the Fig 4 below, the BP GO terms reflect drought-response related terms, such as ‘response to water deprivation’ and ‘response to water’. This shows that drought-response genes were highly enriched. Subsequently, the classification pipeline has the potential to distinguish drought-responsive genes.

**Fig 4:**
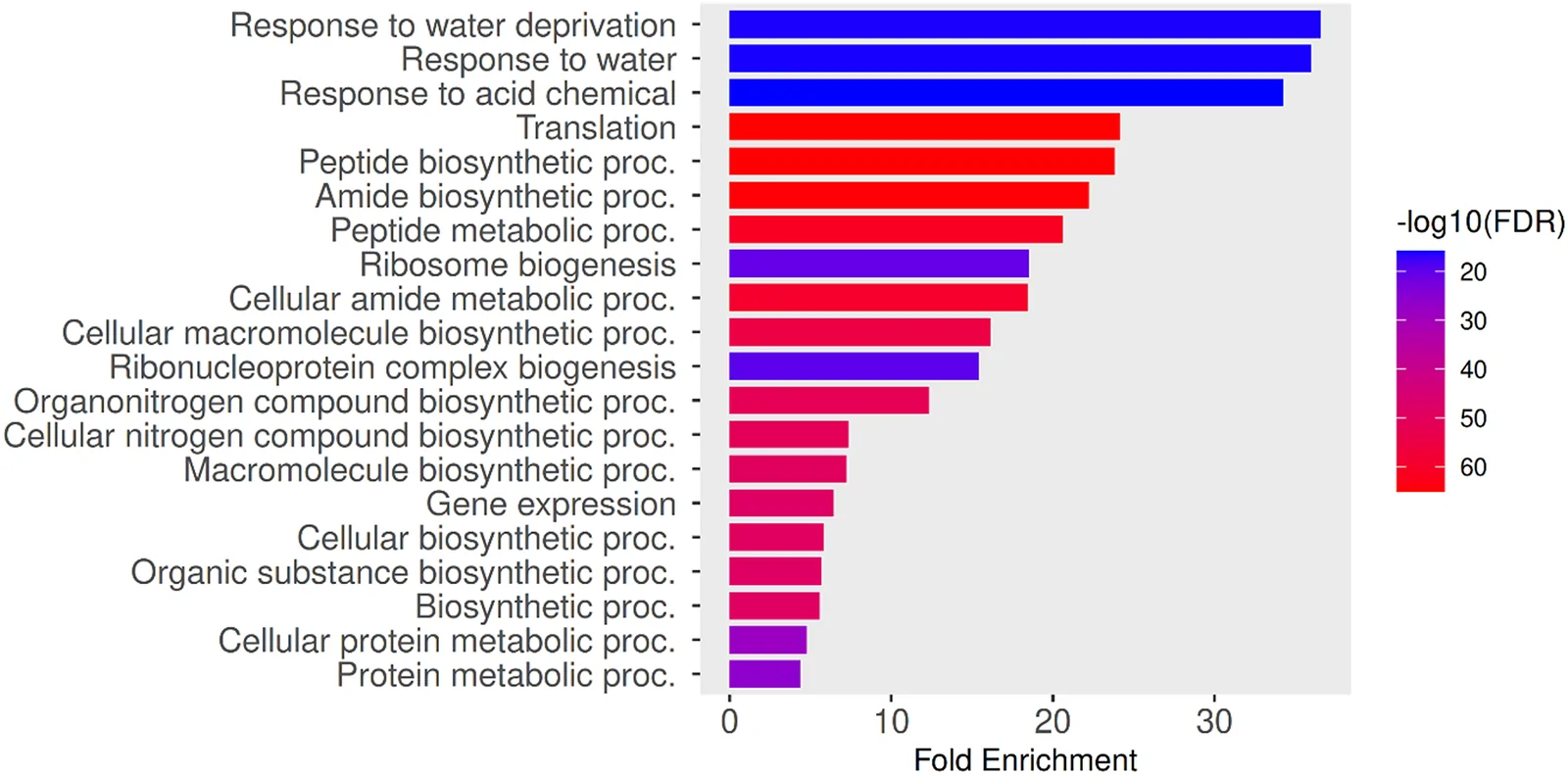
Enriched BP GO terms of the top 5% genes from RF. GO terms associated with drought such as ‘response to water deprivation’ and ‘response to water’ have been enriched in higher folds.

## Discussion

This study proposes an ML framework for classifying drought-responsive genes using multi-omics data. Rice has relatively smaller genome compared wheat and maize and extensive breeding resources [17]. Despite various methods such as ranking, scoring and clustering have been explored, gene-trait association remains treated as a binary classification problem [12]. The difficulty in quantifying the impact of individual genes limits the applicability of regression or clustering algorithms [17]. Consequently, this study used supervised classification algorithms to train the model with existing labelled data. To minimise bias arising from the limited number of experimentally verified genes, as highlighted in previous studies [5,11,12,15], gene labels were obtained from MBKBASE.

Data for 44 features were obtained from multiple omics sources for 2416 genes. QTG-Finder provided 27 features including polymorphism, gene ontology, co-function network, and evolutionary data. Transcriptomics features were incorporated as differential and co-expression analyses have become increasingly important for gene-trait association, particularly through meta-analysis approach [33].

All algorithms struggled with imbalanced class distributions (Datasets 1–5), likely due to overfitting or underfitting. Using *adjusted p-values* to filter non-associated genes improved classification performance while maintaining transparency and avoiding arbitrary data manipulation.

EL methods outperformed DL methods and showed less sensitivity to train-test splits. Model performance was evaluated over 20 iterations with distinct random seeds to ensure robust assessment [46,48]. The fine-tuned RF and XGB models achieved recall of 0.67 and AUC-ROC exceeding 0.87, comparable to or better than previous studies. QTG-Finder reported higher recall (0.79) but lower AUC-ROC (0.73) [5], while GRaiN achieved only 0.41 recall due to false positives from indirect correlations [11]. The improved AUC-ROC supports integrating multi-omics features for enhanced accuracy [12].

Subsequently, assessing the contribution of individual features enhances model interpretability and the reliability of prediction. However, this task is particularly challenging in the context of multi-omics datasets due to their high dimensionality and complexity [15]. In multi-omics systems biology, predicting gene–trait associations for complex polygenic traits relies fundamentally on the non-linear interactions among multiple diverse features rather than isolated inputs. Therefore, feature importance scores were calculated for the EL algorithms, to examine how well these feature coordinate within the broader multi-omics network. The results indicated that transcriptomics-derived features were consistently ranked higher in importance compared to features from other omics sources. Nevertheless, *network_weight* and *paralog_copy_number*, previously identified as key features in QTG-Finder, were ranked 8th and 15th, respectively, in the fine-tuned RF and XGB models.

The addition of new features to the QTG-Finder dataset led to shifts in the ranking of important features for predicting genes associated with abiotic and biotic stress resistance in Arabidopsis. For instance, PPI Count, a feature introduced in an extended version of QTG-Finder, was identified as the third most important feature in an eQTL prediction model developed in a past study [15]. PPI Count captures experimentally verified interaction between genes and their corresponding protein [62]. In the current study, *PPI_Counts_400* and *PPI_Counts_700* also ranked among the top features, reinforcing the value of PPI-based features in the development of omics-driven ML models for gene-trait association studies.

Noteworthy, XGB’s performance relies on aggressive feature selection, which may discard relevant biological signals [63]. In contrast, RF’s bagging approach retains all features, offering more comprehensive and interpretable predictions. Given difficulties distinguishing noise from meaningful signals in multi-omics data [64,65], RF’s inclusive approach may provide more reliable gene classification. As future studies generate integrated omics features from standardized experimental conditions, the resulting data may exhibit stronger relational structures. In such scenarios, architecture designed to capture network-based dependencies, such as graph convolutional networks or autoencoders, could be explored to leverage these inherent relationships.

Nevertheless, the enrichment of drought-related terms further reinforces the potential of RF-based approach. While GO biological process enrichment provides functional validation at the gene level, future studies could extend this framework to pathway-level analysis. This would require modular approaches that account for gene co-expression or correlation networks to avoid upstream biases [66]. Such methods would enable the identification of coordinated gene modules within known drought signalling pathways, including abscisic acid signalling, osmolyte accumulation, and reactive oxygen species scavenging. A pathway-centric classification approach could enhance biological interpretability and reveal regulatory mechanisms underlying drought response. However, this advancement depends on the availability of standardized multi-omics datasets generated under controlled experimental conditions.

Noteworthy, the definition of the negative class relies on current literature annotations and statistical filtering across selected transcriptomic datasets. While our sensitivity analysis across 10 alternative sampled negative sets indicates that performance was not unique to one particular negative sample, the negative class should be viewed as an operational background set rather than a validated negative gold standard. Therefore, all reported metrics are conditional on that operational definition.

The main limitation was incomplete metadata in public repositories, necessitating exclusion of RNA-seq samples. Well-annotated data is essential for advancing data-driven research. This framework can be applied to other traits or species once similar datasets become available. Accurately identifying drought-responsive genes enables breeders to prioritise targets for marker-assisted selection, reducing development time for drought-resistant varieties [67], increasingly urgent as climate change intensifies drought frequency [2].

Overall, ML-based gene-trait analysis is gaining attention in recent studies [5,11,12,15], but application to multi-omics data remains early-stage [68]. Transparent and systematic approaches from dataset curation to performance assessment are essential to ensure reliability and build scientific trust [69].

## Conclusion

Within the present dataset and the fixed, globally constructed feature representation evaluated here, Random Forest achieved the highest and most stable performance among the evaluated models for classifying drought-responsive genes in rice. In this setting, ensemble learning algorithms showed stronger performance than deep learning, highlighting the importance of algorithm selection based on data characteristics rather than model complexity. This study contributes to understanding both the potential and limitations of machine learning in gene-trait association research. However, the analysis focused exclusively on drought response genes due to limited standardized data repositories for other traits. Validation across diverse traits and plant species is essential to determine whether ensemble learning’s advantages persist or whether trait-specific or species-specific factors influence optimal algorithm selection. Establishing standardized multi-omics repositories would accelerate such comparative studies for the development of decision support systems for crop breeding.

## Supporting information

S1 Data The baseline dataset containing 44 features for 2416 stress response genes. (CSV)

S1 Fig Post-diagnostic evaluation of Combat-seq using Principal Component Analysis. (PDF)

S1 Table Description of 44 features and their omics type. (PDF)

S2 Table Performance measures for ML models with Datasets 1–6 and different evaluation approaches. (PDF)

S3 Table Paired t-test results of hyperparameter tuning. (PDF)

S4 Table Feature importance scores from RF and XGB algorithms. (PDF)
