# Supplementary material for: Benchmarking ensemble and deep learning models for drought-responsive gene prioritisation in rice using multi-omics data
Source: PLoS One. 2026 Oct 9;21(10):e0360052. doi: 10.1371/journal.pone.0360052 (PMC13652213; doi:10.1371/journal.pone.0360052)
Supplement: Supplementary file 1 [file pone.0360052.s002.pdf]

Evaluation of Combat-seq

The implementation of *ComBat-seq* to reduce batch-effects (Fig. S1) from the RNA-seq samples of different Bioprojects showed distinct clustering patterns between drought-sensitive and drought-tolerant varieties. This exhibit similarity in gene expressions among drought-tolerant varieities which leads to traits that withstand drought conditions. Stress tolerance is a regulation of specific set of genes (Zhang et al., 2016).

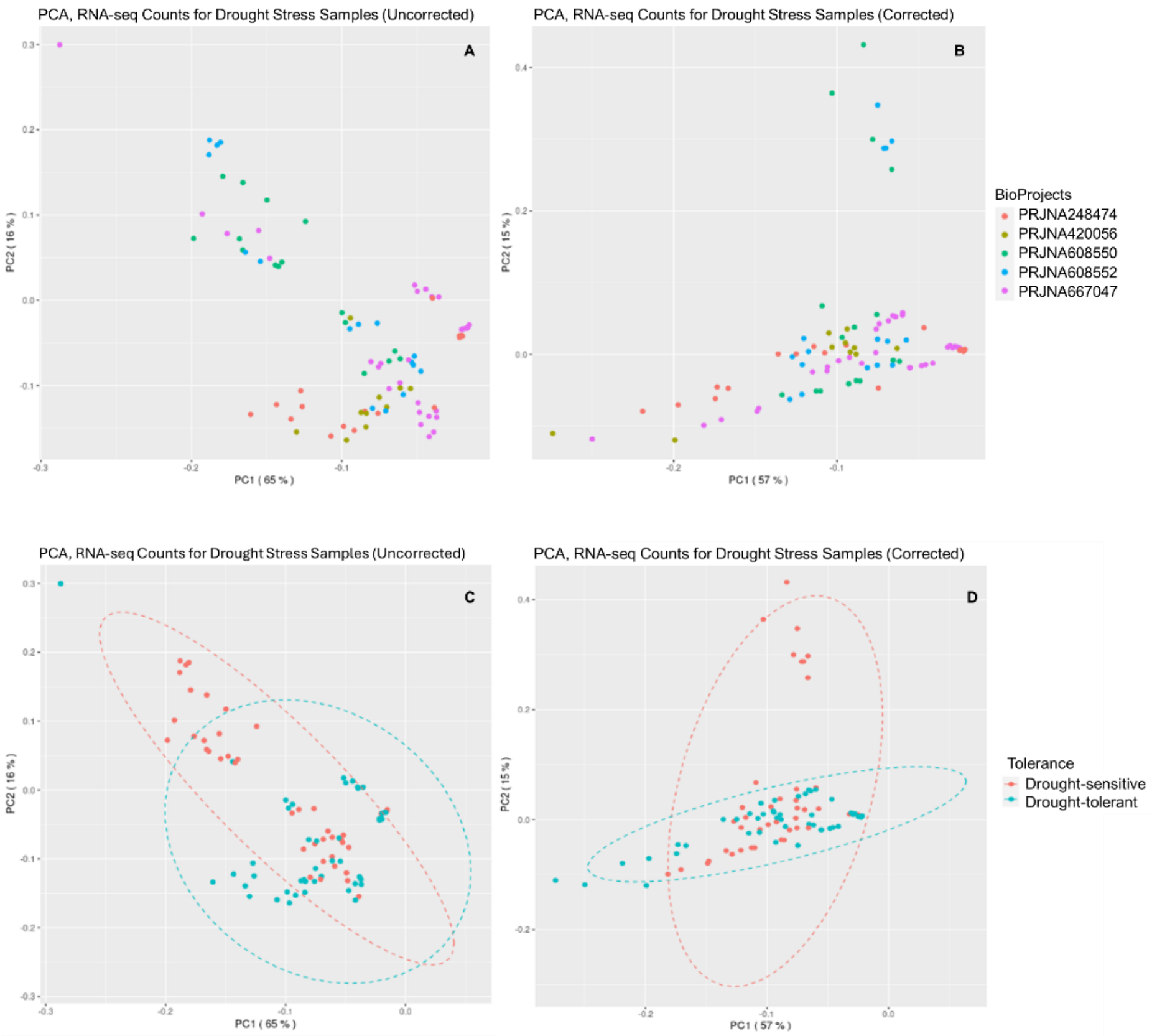

Fig. S1. Principal component analysis (PCA) of rice RNA-seq gene expression profiles before and after batch-effect correction. (A) PCA of uncorrected RNA-seq counts, with samples colour-coded by BioProject. Samples cluster predominantly by BioProject, indicating strong batch effects. (B) PCA of batch-corrected RNA-seq counts, colour-coded by BioProject. Samples are more distributed across BioProjects, demonstrating reduction in batch effects. (C) PCA of uncorrected RNA-seq counts, coloured by drought sensitivity. Clustering is largely influenced by batch rather than biological condition. (D) PCA of batch-corrected RNA-seq counts, coloured by drought sensitivity. Separation between drought-sensitive and drought-tolerant samples along PC1 is more distinct, while some overlap remains due to rice varieties.
