# Supplementary material for: Benchmarking ensemble and deep learning models for drought-responsive gene prioritisation in rice using multi-omics data
Source: PLoS One. 2026 Oct 9;21(10):e0360052. doi: 10.1371/journal.pone.0360052 (PMC13652213; doi:10.1371/journal.pone.0360052)
Supplement: Supplementary file 2 [file pone.0360052.s003.pdf]

Description of omics features.

No.	Features	Descriptions	Rationale
1.	is_start_gained	Binary polymorphism features	Refer to Lin et al., (2019)
2.	is_start_lost	Binary polymorphism features	Refer to Lin et al., (2019)
3.	is_stop_gained	Binary polymorphism features	Refer to Lin et al., (2019)
4.	is_stop_lost	Binary polymorphism features	Refer to Lin et al., (2019)
5.	is_splice_site_SNP	Binary polymorphism features	Refer to Lin et al., (2019)
6.	is_nonsyn_deleterious	The presence of deleterious non-synonymous SNPs in a gene was used as a binary feature	Refer to Lin et al., (2019)
7.	normalized_nonsyn_SNP	Non-synonymous SNPs at conserved protein sequences were predicted to cause deleterious amino acid changes by SIFT4G	Refer to Lin et al., (2019)
8.	is_detoxification_metabolism	Functional gene annotation feature	Refer to Lin et al., (2019)
9.	is_redox_metabolism	Functional gene annotation feature	Refer to Lin et al., (2019)
10.	is_amino_acids_metabolism	Functional gene annotation feature	Refer to Lin et al., (2019)

11.	is_other_metabolism	Functional gene annotation feature	Refer to Lin et al., (2019)
12.	is_macromolecule_metabolism	Functional gene annotation feature	Refer to Lin et al., (2019)
13.	is_TF	Functional gene annotation feature	Refer to Lin et al., (2019)
14.	is_receptor	Functional gene annotation feature	Refer to Lin et al., (2019)
15.	is_kinase	Functional gene annotation feature	Refer to Lin et al., (2019)
16.	is_transporter	Functional gene annotation feature	Refer to Lin et al., (2019)
17.	is_carbohydrates_metabolism	Functional gene annotation feature	Refer to Lin et al., (2019)
18.	is_nucleotides_metabolism	Functional gene annotation feature	Refer to Lin et al., (2019)
19.	is_energy_metabolism	Functional gene annotation feature	Refer to Lin et al., (2019)
20.	is_fatty_acids_lipids_metabolism	Functional gene annotation feature	Refer to Lin et al., (2019)
21.	is_specialized_metabolism	Functional gene annotation feature	Refer to Lin et al., (2019)
22.	is_cofactors_metabolism	Functional gene annotation feature	Refer to Lin et al., (2019)
23.	is_hormones_metabolism	Functional gene annotation feature	Refer to Lin et al., (2019)
24.	is_inorganic_nutrients_metabolism	Functional gene annotation feature	Refer to Lin et al., (2019)
25.	paralog_copy_number	Paralog copy number	Refer to Lin et al., (2019)

26.	is_essential_gene	essential gene prediction	Refer to Lin et al., (2019)
27.	network_weight	The sum of all the edge weights from gene network.	Refer to Lin et al., (2019)
28.	eQTL	Genes of cis expression quantitative trait loci group containing SNPs within promoter region.	Functional single nucleotide polymorphisms (SNPs) within cis-regulatory promoter regions directly alter transcription factor binding affinities, linking genetic polymorphisms to structural changes in stress-responsive gene expression traits (House et al., 2014).
29.	Basemean_Tol	Average of the normalized count values, dividing by size factors, taken from drought tolerant samples.	Establishes the overall transcript abundance of a gene across all drought tolerant samples.
30.	Log2FC_Deseq2_Tol	Magnitude of change in gene expression level in drought tolerant samples.	Differential expression magnitude identifies genes actively responding to environmental triggers; high log2 fold-changes pinpoint key

			drivers of downstream stress adaptation (Love et al., 2014).
31.	Up_Tol	Upregulated genes in tolerant samples.	Helps isolate genes overexpressed leading tolerance pathways under drought treatment,
32.	Dn_Tol	Downregulated genes in tolerant samples.	Helps isolate genes underexpressed leading tolerance pathways under drought treatment,
33.	Basemean_Sen	Average of the normalized count values, dividing by size factors, taken from drought sensitive samples.	Establishes the overall transcript abundance of a gene across all drought sensitive samples.
34.	Log2FC_Deseq2_Sen	Magnitude of change in gene expression level in sensitive samples.	Differential expression magnitude identifies genes actively responding to environmental triggers; high log2 fold-changes pinpoint key drivers of downstream stress adaptation (Love et al., 2014).

35.	Up_Sen	Upregulated genes in drought sensitive samples.	Helps isolate genes overexpressed leading susceptibility pathways under drought treatment,
36.	Dn_Sen	Downregulated genes in drought sensitive samples.	Helps isolate genes underexpressed leading susceptibility pathways under drought treatment.
37.	Mod_No_Tol	WGCNA module numbers generated using tolerant samples	Network modules represent functional units of co-regulated genes. Grouping genes captures system-level biological processes rather than isolated single-gene events (Langfelder and Horvath, 2008).
38.	Mod_High_Corr_Tol	Highly correlated modules with drought tolerant traits	Pinpoints networks driven by stress response, and physiological traits unique to the tolerant genotype.

39.	Hub_Genes_Tol	Genes from tolerant samples belonging to module correlation > 0.5, and achieves GS > 0.3 and MM > 0.7	Hub genes possess high Module Membership (MM) and Gene Significance (GS). They act as central regulatory nodes; perturbing them typically compromises the stability of the entire stress-response network, especially for tolerant traits.
40.	Mod_No_Sen	WGCNA module numbers generated using sensitive samples	Network modules represent functional units of co-regulated genes. Grouping genes captures system-level biological processes rather than isolated single-gene events (Langfelder and Horvath, 2008).
41.	Mod_High_Corr_Sen	Highly correlated modules with drought sensitive traits	Pinpoints networks driven by stress response, and physiological traits unique to the sensitive genotype.

42.	Hub_Genes_Sen	Genes from sensitive samples belonging to module correlation > 0.5, and achieves GS > 0.4 and MM> 0.8	Hub genes act as central regulatory nodes; perturbing them typically compromises the stability of the entire susceptible stress-response network.
43.	PPI_counts_400	Number of genes with PPI scores above 400, reflecting moderate connection	Protein-Protein Interaction (PPI) networks map physical functional complexes. Moderate-confidence interactions capture broader, transient signaling cascades and regulatory pathways (Szklarczyk et al., 2021).
44.	PPI_counts_700	Number of genes with PPI scores above 700, reflecting strong connection	High-confidence interactions may filter out false positives, isolating stable interactions.

Note: Each colour represents an omic type as shown in the table below.

(green)	Genomic Features
(blue)	Transcriptomics Features
(pink)	Proteomics Features
