# Supplementary material for: Benchmarking ensemble and deep learning models for drought-responsive gene prioritisation in rice using multi-omics data
Source: PLoS One. 2026 Oct 9;21(10):e0360052. doi: 10.1371/journal.pone.0360052 (PMC13652213; doi:10.1371/journal.pone.0360052)
Supplement: Supplementary file 3 [file pone.0360052.s004.pdf]

Performance of Random Forest

Table A Performance of RF with NCV

Dataset	Accuracy	Precision	Recall	F1-Score	AUC-ROC
DS1	0.87 ± 0.00	0.81 ± 0.03	0.10 ± 0.01	0.18 ± 0.02	0.67 ± 0.01
DS2	0.82 ± 0.00	0.89 ± 0.02	0.14 ± 0.01	0.24 ± 0.01	0.72 ± 0.01
DS3	0.77 ± 0.00	0.92 ± 0.01	0.16 ± 0.00	0.27 ± 0.01	0.79 ± 0.01
DS4	0.73 ± 0.00	0.92 ± 0.02	0.20 ± 0.01	0.32 ± 0.02	0.82 ± 0.00
DS5	0.80 ± 0.01	0.96 ± 0.01	0.50 ± 0.03	0.66 ± 0.02	0.85 ± 0.00
DS6	0.81 ± 0.01	0.92 ± 0.01	0.62 ± 0.02	0.74 ± 0.01	0.87 ± 0.00

Table B Performance stability of RF

Approach	Accuracy	Precision	Recall	F1-Score	AUC-ROC
NCV	0.81 ± 0.01	0.92 ± 0.01	0.62 ± 0.02	0.74 ± 0.01	0.87 ± 0.00
CV	0.82 ± 0.00	0.91 ± 0.01	0.63 ± 0.01	0.74 ± 0.01	0.86 ± 0.00
Holdout	0.82 ± 0.03	0.91 ± 0.06	0.64 ± 0.07	0.75 ± 0.05	0.87 ± 0.02

Performance of XGBoost

Table A Performance of XGB with NCV

Dataset	Accuracy	Precision	Recall	F1-Score	AUC-ROC
DS1	0.86 ± 0.00	0.54 ± 0.04	0.16 ± 0.01	0.24 ± 0.01	0.67 ± 0.01
DS2	0.81 ± 0.00	0.60 ± 0.03	0.29 ± 0.02	0.39 ± 0.02	0.73 ± 0.01
DS3	0.83 ± 0.01	0.77 ± 0.02	0.53 ± 0.01	0.63 ± 0.01	0.80 ± 0.01
DS4	0.80 ± 0.01	0.76 ± 0.02	0.60 ± 0.01	0.67 ± 0.01	0.82 ± 0.01
DS5	0.80 ± 0.01	0.78 ± 0.02	0.66 ± 0.01	0.71 ± 0.01	0.84 ± 0.01
DS6	0.79 ± 0.01	0.79 ± 0.01	0.70 ± 0.01	0.74 ± 0.01	0.86 ± 0.01

Table B Performance stability of XGB

Approach	Accuracy	Precision	Recall	F1-Score	AUC-ROC
NCV	0.79 ± 0.01	0.79 ± 0.01	0.70 ± 0.01	0.74 ± 0.01	0.86 ± 0.01
CV	0.77 ± 0.00	0.75 ± 0.00	0.69 ± 0.00	0.72 ± 0.00	0.85 ± 0.00
Holdout	0.79 ± 0.02	0.80 ± 0.06	0.71 ± 0.02	0.75 ± 0.03	0.85 ± 0.02

Performance of 1D-CNN

Table A Performance of 1D-CNN with different architectures

**No.of Layers**								
**MP-Conv (pairs)**	**Dropout**	Activation	Layers Units	**Accuracy**	**Precision**	**Recall**	**F1-Score**	**AUC-ROC**
1	-	sigmoid	64	0.64 ± 0.02	0.65 ± 0.04	0.48 ± 0.09	0.50 ± 0.06	0.67 ± 0.02
1	-	softmax	64	0.62 ± 0.02	0.62 ± 0.05	0.42 ± 0.03	0.50 ± 0.03	0.60 ± 0.02
1	-	sigmoid	32	0.62 ± 0.05	0.60 ± 0.12	0.41 ± 0.19	0.45 ± 0.13	0.62 ± 0.11
1	1	sigmoid	64	0.62 ± 0.05	0.60 ± 0.02	0.49 ± 0.20	0.52 ± 0.09	0.62 ± 0.04
**1**	**2**	**sigmoid**	**64**	**0.62 ± 0.04**	**0.57 ± 0.06**	**0.56 ± 0.20**	**0.54 ± 0.11**	**0.69 ± 0.02**
2	-	sigmoid	64;128	0.69 ± 0.05	0.62 ± 0.05	0.44 ± 0.24	0.49 ± 0.18	0.61 ± 0.55
2	1	sigmoid	64;128	0.67 ± 0.20	0.61 ± 0.12	0.47 ± 0.19	0.50 ± 0.10	0.60 ± 0.26
2	2	sigmoid	64;128	0.73 ± 0.01	0.64 ± 0.02	0.47 ± 0.03	0.53 ± 0.02	0.55 ± 0.03
3	-	sigmoid	64;128;64	0.68 ± 0.09	0.61 ± 0.02	0.44 ± 0.11	0.50 ± 0.07	0.53 ± 0.06
3	3	sigmoid	64;128;64	0.65 ± 0.02	0.64 ± 0.08	0.42 ± 0.08	0.44 ± 0.05	0.52 ± 0.13

Table B Performance of 1D-CNN with NCV

**Dataset**	**Accuracy**	**Precision**	**Recall**	**F1-Score**	**AUC-ROC**
DS1	0.85 ± 0.02	0.39 ± 0.16	0.08 ± 0.03	0.10 ± 0.02	0.60 ± 0.01
DS2	0.78 ± 0.03	0.55 ± 0.19	0.14 ± 0.07	0.17 ± 0.07	0.61 ± 0.02
DS3	0.70 ± 0.04	0.56 ± 0.10	0.29 ± 0.12	0.31 ± 0.06	0.62 ± 0.01
DS4	0.68 ± 0.02	0.63 ± 0.10	0.32 ± 0.12	0.36 ± 0.07	0.64 ± 0.03
DS5	0.65 ± 0.02	0.64 ± 0.08	0.42 ± 0.08	0.44 ± 0.05	0.65 ± 0.02
DS6	0.62 ± 0.04	0.57 ± 0.06	0.56 ± 0.20	0.54 ± 0.11	0.69 ± 0.02

Table C Performance stability of 1D-CNN

**Approach**	**Accuracy**	**Precision**	**Recall**	**F1-Score**	**AUC-ROC**
NCV	0.62 ± 0.04	0.57 ± 0.06	0.56 ± 0.20	0.54 ± 0.11	0.69 ± 0.02
CV	0.81 ± 0.01	0.92 ± 0.02	0.62 ± 0.02	0.74 ± 0.01	0.79 ± 0.01
Holdout	0.67 ± 0.03	0.64 ± 0.08	0.53 ± 0.28	0.57 ± 0.10	0.70 ± 0.06

Performance of MLP

Table A Performance of MLP with different architectures

No.of Layers						
Dense	Dropout	Accuracy	Precision	Recall	F1-Score	AUC-ROC
3	-	0.64 ± 0.03	0.65 ± 0.05	0.44 ± 0.18	0.43 ± 0.11	0.62 ± 0.06
3	1	0.62 ± 0.02	0.66 ± 0.05	0.45 ± 0.13	0.46 ± 0.07	0.62 ± 0.03
**3**	**2**	**0.64 ± 0.01**	**0.60 ± 0.01**	**0.50 ± 0.10**	**0.52 ± 0.05**	**0.64 ± 0.02**
4	-	0.63 ± 0.02	0.61 ± 0.04	0.44 ± 0.07	0.52 ± 0.03	0.64 ± 0.02
4	1	0.63 ± 0.02	0.61 ± 0.03	0.43 ± 0.05	0.52 ± 0.05	0.64 ± 0.03
4	2	0.66 ± 0.04	0.65 ± 0.10	0.49 ± 0.16	0.51 ± 0.13	0.65 ± 0.08
4	3	0.62 ± 0.02	0.58 ± 0.03	0.52 ± 0.07	0.52 ± 0.03	0.63 ± 0.02
5	-	0.64 ± 0.03	0.63 ± 0.06	0.44 ± 0.08	0.43 ± 0.10	0.60 ± 0.03

Table B Performance of MLP with NCV

Dataset	Accuracy	Precision	Recall	F1-Score	AUC-ROC
DS1	0.81 ± 0.03	0.40 ± 0.06	0.19 ± 0.07	0.19 ± 0.04	0.54 ± 0.02
DS2	0.76 ± 0.03	0.53 ± 0.09	0.27 ± 0.07	0.29 ± 0.03	0.59 ± 0.02
DS3	0.71 ± 0.03	0.59 ± 0.07	0.30 ± 0.07	0.33 ± 0.03	0.59 ± 0.01
DS4	0.64 ± 0.04	0.55 ± 0.08	0.36 ± 0.13	0.40 ± 0.05	0.60 ± 0.02
DS5	0.65 ± 0.03	0.63 ± 0.04	0.42 ± 0.09	0.42 ± 0.05	0.61 ± 0.03
DS6	0.66 ± 0.04	0.65 ± 0.10	0.49 ± 0.26	0.51 ± 0.17	0.65 ± 0.08

Table C Performance stability of MLP

Approach	Accuracy	Precision	Recall	F1-Score	AUC-ROC
NCV	0.66 ± 0.04	0.65 ± 0.10	0.49 ± 0.26	0.51 ± 0.17	0.65 ± 0.08
CV	0.63 ± 0.10	0.62 ± 0.04	0.57 ± 0.09	0.52 ± 0.04	0.66 ± 0.04
Holdout	0.62 ± 0.04	0.59 ± 0.12	0.44 ± 0.22	0.46 ± 0.13	0.61 ± 0.06

Performance of Logistic Regression

Table A Performance of LR with NCV

Dataset	Accuracy	Precision	Recall	F1-Score	AUC-ROC
DS1	0.87 ± 0.00	0.73 ± 0.05	0.09 ± 0.01	0.16 ± 0.02	0.59 ± 0.01
DS2	0.81 ± 0.00	0.79 ± 0.02	0.14 ± 0.01	0.23 ± 0.02	0.56 ± 0.00
DS3	0.76 ± 0.00	0.77 ± 0.02	0.16 ± 0.00	0.26 ± 0.00	0.59 ± 0.00
DS4	0.71 ± 0.00	0.80 ± 0.03	0.16 ± 0.00	0.27 ± 0.00	0.60 ± 0.01
DS5	0.67 ± 0.00	0.77 ± 0.03	0.18 ± 0.01	0.29 ± 0.01	0.61 ± 0.00
DS6	0.63 ± 0.00	0.73 ± 0.03	0.20 ± 0.01	0.31 ± 0.01	0.63 ± 0.01

Table B Performance stability of LR

Approach	Accuracy	Precision	Recall	F1-Score	AUC-ROC
NCV	0.67 ± 0.01	0.75 ± 0.03	0.33 ± 0.01	0.46 ± 0.01	0.75 ± 0.01
CV	0.78 ± 0.00	0.78 ± 0.00	0.66 ± 0.00	0.72 ± 0.00	0.81 ± 0.00
Holdout	0.77 ± 0.04	0.79 ± 0.06	0.64 ± 0.06	0.70 ± 0.05	0.81 ± 0.04

Performance of Support Vector Machine

Table A Performance of SVM with NCV (kernel='rbf')

Dataset	Accuracy	Precision	Recall	F1-Score	AUC-ROC
DS1	0.87 ± 0.00	0.58 ± 0.04	0.13 ± 0.01	0.21 ± 0.02	0.60 ± 0.01
DS2	0.81 ± 0.00	0.69 ± 0.04	0.17 ± 0.01	0.27 ± 0.01	0.68 ± 0.01
DS3	0.76 ± 0.00	0.72 ± 0.03	0.19 ± 0.01	0.30 ± 0.02	0.74 ± 0.01
DS4	0.72 ± 0.00	0.74 ± 0.01	0.22 ± 0.01	0.33 ± 0.01	0.75 ± 0.01
DS5	0.69 ± 0.01	0.78 ± 0.03	0.28 ± 0.01	0.41 ± 0.01	0.75 ± 0.01
DS6	0.67 ± 0.01	0.75 ± 0.03	0.33 ± 0.01	0.46 ± 0.01	0.75 ± 0.01

Table B Performance stability of SVM

Approach	Accuracy	Precision	Recall	F1-Score	AUC-ROC
NCV	0.63 ± 0.00	0.73 ± 0.03	0.20 ± 0.01	0.31 ± 0.01	0.63 ± 0.01
CV	0.62 ± 0.00	0.66 ± 0.01	0.23 ± 0.01	0.34 ± 0.01	0.61 ± 0.00
Holdout	0.63 ± 0.04	0.91 ± 0.10	0.14 ± 0.04	0.25 ± 0.05	0.63 ± 0.04

Performance of Naïve Bayes

Table A Performance of NB with NCV

Dataset	Accuracy	Precision	Recall	F1-Score	AUC-ROC
DS1	0.81 ± 0.00	0.32 ± 0.01	0.30 ± 0.00	0.31 ± 0.00	0.67 ± 0.00
DS2	0.77 ± 0.00	0.43 ± 0.02	0.27 ± 0.01	0.33 ± 0.01	0.71 ± 0.01
DS3	0.75 ± 0.00	0.58 ± 0.00	0.32 ± 0.01	0.41 ± 0.01	0.74 ± 0.00
DS4	0.73 ± 0.00	0.66 ± 0.02	0.38 ± 0.01	0.48 ± 0.01	0.76 ± 0.00
DS5	0.73 ± 0.01	0.74 ± 0.02	0.47 ± 0.02	0.57 ± 0.02	0.78 ± 0.01
DS6	0.73 ± 0.01	0.76 ± 0.01	0.53 ± 0.02	0.62 ± 0.02	0.78 ± 0.00

Table B Performance stability of NB

Approach	Accuracy	Precision	Recall	F1-Score	AUC-ROC
NCV	0.73 ± 0.01	0.76 ± 0.01	0.53 ± 0.02	0.62 ± 0.02	0.78 ± 0.00
CV	0.73 ± 0.00	0.79 ± 0.00	0.51 ± 0.00	0.62 ± 0.00	0.78 ± 0.00
Holdout	0.73 ± 0.04	0.77 ± 0.06	0.53 ± 0.07	0.62 ± 0.06	0.78 ± 0.04

Performance of K-Nearest Neighbour

Table A Performance of KNN with NCV

Dataset	Accuracy	Precision	Recall	F1-Score	AUC-ROC
DS1	0.86 ± 0.00	0.50 ± 0.04	0.14 ± 0.01	0.22 ± 0.01	0.57 ± 0.01
DS2	0.79 ± 0.00	0.47 ± 0.02	0.19 ± 0.01	0.27 ± 0.01	0.59 ± 0.01
DS3	0.71 ± 0.01	0.45 ± 0.02	0.24 ± 0.02	0.30 ± 0.02	0.60 ± 0.01
DS4	0.66 ± 0.01	0.49 ± 0.02	0.32 ± 0.01	0.38 ± 0.01	0.64 ± 0.01
DS5	0.63 ± 0.01	0.53 ± 0.02	0.40 ± 0.01	0.45 ± 0.01	0.65 ± 0.01
DS6	0.61 ± 0.01	0.55 ± 0.02	0.48 ± 0.02	0.51 ± 0.02	0.67 ± 0.01

Table B Performance stability of KNN

Approach	Accuracy	Precision	Recall	F1-Score	AUC-ROC
NCV	0.61 ± 0.01	0.55 ± 0.02	0.48 ± 0.02	0.51 ± 0.02	0.67 ± 0.01
CV	0.58 ± 0.00	0.51 ± 0.00	0.48 ± 0.00	0.49 ± 0.00	0.62 ± 0.00
Holdout	0.60 ± 0.04	0.53 ± 0.07	0.48 ± 0.05	0.50 ± 0.05	0.65 ± 0.03
