# Supplementary material for: Benchmarking ensemble and deep learning models for drought-responsive gene prioritisation in rice using multi-omics data
Source: PLoS One. 2026 Oct 9;21(10):e0360052. doi: 10.1371/journal.pone.0360052 (PMC13652213; doi:10.1371/journal.pone.0360052)
Supplement: Supplementary file 4 [file pone.0360052.s005.pdf]

**Random Forest**		
**Default Paraemeters vs Fine-tuned Parameters**		
**Accuracy**		
t-Test: Paired Two Sample for Means		
	Variable 1	*Variable 2*
Mean	0.8143719	0.8240428
Variance	0.000035798391	0.000034690347
Observations	20	20
Pearson Correlat	0.2291451991	
Hypothesized Me	0	
df	19	
t Stat	-5.867157596	
P(T<=t) one-tail	0.000005958724	
t Critical one-tail	1.729132793	
P(T<=t) two-tail	0.000011917449	
t Critical two-tail	2.093024022	
**Precision**		
t-Test: Paired Two Sample for Means		
	Variable 1	*Variable 2*
Mean	0.92036235	0.89239655
Variance	0.000170276056	0.000127489292
Observations	20	20
Pearson Correlat	0.5308104777	
Hypothesized Me	0	
df	19	
t Stat	10.51953203	
P(T<=t) one-tail	0.000000001156	
t Critical one-tail	1.729132793	
P(T<=t) two-tail	0.000000002313	
t Critical two-tail	2.093024022	
**Recall**		
t-Test: Paired Two Sample for Means		
	Variable 1	*Variable 2*
Mean	0.6227608	0.67113305
Variance	0.000225673062	0.000067963588
Observations	20	20
Pearson Correlat	0.00657518963	
Hypothesized Me	0	
df	19	
t Stat	-12.65942206	

P(T<=t) one-tail	0	
t Critical one-tail	1.729132793	
P(T<=t) two-tail	0.000000000104	
t Critical two-tail	2.093024022	

F1-score

t-Test: Paired Two Sample for Means

	Variable 1	*Variable 2*
Mean	0.74025765	0.76439155
Variance	0.000088267076	0.000060620659
Observations	20	20
Pearson Correlat	-0.01960579461	
Hypothesized Me	0	
df	19	
t Stat	-8.761319607	
P(T<=t) one-tail	0.000000021170	
t Critical one-tail	1.729132793	
P(T<=t) two-tail	0.000000042341	
t Critical two-tail	2.093024022	

AUC-ROC

t-Test: Paired Two Sample for Means

	Variable 1	*Variable 2*
Mean	0.87146565	0.869134
Variance	0.000013362273	0.000016618223
Observations	20	20
Pearson Correlat	0.6408733412	
Hypothesized Me	0	
df	19	
t Stat	3.161223069	
P(T<=t) one-tail	0.00257092595	
t Critical one-tail	1.729132793	
P(T<=t) two-tail	0.0051418519	
t Critical two-tail	2.093024022	

**XGBoost**		
**Default Paraemeters vs Fine-tuned Parameters**		
**Accuracy**		
t-Test: Paired Two Sample for Means		
	Variable 1	*Variable 2*
Mean	0.79039545	0.80935345
Variance	0.000046974421	0.000039561739
Observations	20	20
Pearson Correlat	0.2558726368	
Hypothesized Me	0	
df	19	
t Stat	-10.55870839	
P(T<=t) one-tail	0.000000001088	
t Critical one-tail	1.729132793	
P(T<=t) two-tail	0.000000002176	
t Critical two-tail	2.093024022	
**Precision**		
t-Test: Paired Two Sample for Means		
	Variable 1	*Variable 2*
Mean	0.79013515	0.85564405
Variance	0.000126138936	0.000176319677
Observations	20	20
Pearson Correlat	0.1205607245	
Hypothesized Me	0	
df	19	
t Stat	-17.94598206	
P(T<=t) one-tail	0	
t Critical one-tail	1.729132793	
P(T<=t) two-tail	0	
t Critical two-tail	2.093024022	
**Recall**		
t-Test: Paired Two Sample for Means		
	Variable 1	*Variable 2*
Mean	0.6974011	0.66923505
Variance	0.000153627577	0.000074137774
Observations	20	20
Pearson Correlat	0.4645699021	
Hypothesized Me	0	
df	19	
t Stat	11.10736395	

P(T<=t) one-tail	0.000000000472	
t Critical one-tail	1.729132793	
P(T<=t) two-tail	0.000000000944	
t Critical two-tail	2.093024022	

F1-score

t-Test: Paired Two Sample for Means

	Variable 1	*Variable 2*
Mean	0.73901165	0.74929535
Variance	0.000075497445	0.000059248864
Observations	20	20
Pearson Correlat	0.1590171775	
Hypothesized Me	0	
df	19	
t Stat	-4.317307511	
P(T<=t) one-tail	0.000185720604	
t Critical one-tail	1.729132793	
P(T<=t) two-tail	0.000371441207	
t Critical two-tail	2.093024022	

AUC-ROC

t-Test: Paired Two Sample for Means

	Variable 1	*Variable 2*
Mean	0.85661675	0.86179375
Variance	0.000042675497	0.000012743933
Observations	20	20
Pearson Correlat	0.422728715	
Hypothesized Me	0	
df	19	
t Stat	-3.874735371	
P(T<=t) one-tail	0.000509999352	
t Critical one-tail	1.729132793	
P(T<=t) two-tail	0.001019998705	
t Critical two-tail	2.093024022	
