# Supplementary material for: Benchmarking ensemble and deep learning models for drought-responsive gene prioritisation in rice using multi-omics data
Source: PLoS One. 2026 Oct 9;21(10):e0360052. doi: 10.1371/journal.pone.0360052 (PMC13652213; doi:10.1371/journal.pone.0360052)
Supplement: Supplementary file 5 [file pone.0360052.s006.pdf]

Feature importance score from Random Forest with default parameters.

**Features**	**Scores**
DN_Sen	0.1385504
baseMean_Tol	0.117052807
baseMean_Sen	0.1127859205
log2FC_DESeq2	0.101841205
PPI.Counts_400	0.0874294415
PPI.Count_700	0.0551740265
DN_Tol	0.050900874
network_weight	0.047572787
log2FC_DESeq2	0.046881562
UP_Tol	0.0438540895
hub_genes_dr_S	0.0376344435
mod_high_corr_c	0.0257883
UP_Sen	0.0251037405
paralog_copy_nu	0.020664094
mod_no_Sen	0.020260252
is_inorganic_nutr	0.0192333235
normalized_nons	0.00936836385
mod_no_Tol	0.0084732109
is_carbohydrates	0.00373311291
is_nonsyn_delete	0.00279228835
is_stop_gained	0.00252888765
is_essential_gen	0.002118196385
hub_genes_dr_T	0.001831840748
is_TF	0.0015724859
is_specialized_m	0.00154800195
is_hormones_me	0.00144357415
mod_high_corr_c	0.0013470974
is_kinase	0.001113617658
is_energy_metab	0.001090950485
is_detoxification_	0.001086085306

Feature importance score from Random Forest with default parameters.				
**Features**	**Scores**			
eQTL	0.0010800484			
is_start_gained	0.000988556315			
is_fatty_acids_lip	0.000943746865			
is_other_metabo	0.00091073382			
is_transporter	0.000903184025			
is_amino_acids_	0.000839643745			
is_cofactors_met	0.000733512487			
is_macromolecul	0.00071170221			
is_redox_metabo	0.0005777729			
is_nucleotides_m	0.000389743501			
is_receptor	0.0003825			
is_start_lost	0.00035575			
is_splice_site_SN	0.00030782419			
is_stop_lost	0.00009975			

Feature importance score from Random Forest with fine-tuned parameters.				
**Features**	**Scores**			
log2FC_DESeq2	0.1132015			
baseMean_Sen	0.11282025			
baseMean_Tol	0.1091621			
log2FC_DESeq2	0.0891031			
DN_Sen	0.08587205			
network_weight	0.04840835			
UP_Tol	0.046412			
paralog_copy_nu	0.0461265			
PPI.Counts_400	0.0442645			
UP_Sen	0.03825585			
normalized_nons	0.03404735			
DN_Tol	0.0298315			
PPI.Count_700	0.02861275			
hub_genes_dr_S	0.02310885			
mod_no_Sen	0.0193036			
mod_high_corr_c	0.0182895			
is_inorganic_nutr	0.0140282			
mod_no_Tol	0.00918695			
is_specialized_m	0.00680145			
eQTL	0.0066655			
is_nonsyn_delete	0.0063039			
is_essential_gen	0.00582495			
is_TF	0.0057657			
is_stop_gained	0.0057646			
is_kinase	0.0057466			
is_start_gained	0.0051226			
is_macromolecul	0.00492125			
is_carbohydrates	0.0048214			
is_hormones_me	0.0041115			
is_transporter	0.0038837			

Feature importance score from Random Forest with fine-tuned parameters.

Features	Scores			
is_other_metabo	0.0033247			
is_amino_acids_	0.0029377			
hub_genes_dr_T	0.00271015			
mod_high_corr_c	0.0027081			
is_fatty_acids_lip	0.0025202			
is_cofactors_met	0.00228225			
is_energy_metab	0.00202785			
is_detoxification_	0.0014082			
is_redox_metabo	0.00138585			
is_splice_site_SN	0.00063295			
is_receptor	0.00063225			
is_stop_lost	0.00062345			
is_nucleotides_m	0.0006202			
is_start_lost	0.000418			

Feature importance score from XGBoost with default parameters.

Features	Scores			
UP_Tol	0.18060535			
DN_Sen	0.16277915			
UP_Sen	0.1208792			
is_inorganic_nutr	0.04230205			
hub_genes_dr_S	0.04056435			
baseMean_Tol	0.03793475			
PPI.Count_700	0.0354392			
DN_Tol	0.0318943			
baseMean_Sen	0.02542815			
PPI.Counts_400	0.02424215			
is_stop_gained	0.02100065			
mod_high_corr_c	0.0204168			
log2FC_DESeq2	0.01782695			
log2FC_DESeq2	0.01691755			
paralog_copy_nu	0.0146047			
mod_no_Tol	0.01323405			
is_kinase	0.0130789			
is_essential_gen	0.01248805			
is_specialized_m	0.0123927			
is_carbohydrates	0.0123809			
is_detoxification_	0.011922			
is_macromolecul	0.0118624			
network_weight	0.01175425			
mod_no_Sen	0.01165745			
normalized_nons	0.0114472			
is_TF	0.0114171			
is_nonsyn_delete	0.01089975			
eQTL	0.0107703			
is_start_gained	0.0102673			
is_hormones_me	0.0089952			

Feature importance score from XGBoost with default parameters.

Features	Scores			
is_amino_acids_	0.00875465			
is_transporter	0.0053537			
mod_high_corr_c	0.0041652			
is_other_metabo	0.0039487			
is_energy_metab	0.00324285			
hub_genes_dr_T	0.0030691			
is_nucleotides_m	0.0020638			
is_fatty_acids_lip	0.0016681			
is_cofactors_met	0.00033085			
is_redox_metabo	0			
is_receptor	0			
is_start_lost	0			
is_stop_lost	0			
is_splice_site_SN	0			

Feature importance score from XGBoost with fine-tuned parameters.

Features	Scores
UP_Sen	0.16734
UP_Tol	0.1584683
DN_Sen	0.1488415
baseMean_Tol	0.0764751
PPI.Count_700	0.04804115
hub_genes_dr_S	0.043665
baseMean_Sen	0.04263385
PPI.Counts_400	0.02929245
log2FC_DESeq2	0.0272564
DN_Tol	0.0272156
is_inorganic_nutr	0.02587025
log2FC_DESeq2	0.024549
mod_high_corr_c	0.0193191
paralog_copy_nu	0.0170215
is_specialized_m	0.01456095
is_stop_gained	0.01311035
network_weight	0.01141945
normalized_nons	0.0109409
is_nonsyn_delete	0.01066915
is_carbohydrates	0.0096068
mod_no_Sen	0.00948505
is_essential_gen	0.0075827
is_kinase	0.0070996
is_detoxification_	0.0068988
is_macromolecul	0.0063409
mod_no_Tol	0.0061195
is_TF	0.0060819
is_hormones_me	0.00589865
eQTL	0.005192
is_transporter	0.0035613

Feature importance score from XGBoost with fine-tuned parameters.

Features	Scores			
is_amino_acids_	0.00350125			
is_other_metabo	0.00301525			
is_start_gained	0.0018667			
is_energy_metab	0.0010594			
mod_high_corr_c	0			
hub_genes_dr_T	0			
is_nucleotides_m	0			
is_fatty_acids_lip	0			
is_cofactors_met	0			
is_redox_metabo	0			
is_receptor	0			
is_start_lost	0			
is_stop_lost	0			
is_splice_site_SN	0			
